# Pressure and Temperature Dependence of Local Structure and Dynamics in an Ionic Liquid

**DOI:** 10.1021/acs.jpcb.1c00147

**Published:** 2021-03-03

**Authors:** Filippa Lundin, Henriette Wase Hansen, Karolina Adrjanowicz, Bernhard Frick, Daniel Rauber, Rolf Hempelmann, Olga Shebanova, Kristine Niss, Aleksandar Matic

**Affiliations:** † Department of Physics, Chalmers University of Technology, SE-41296 Göteborg, Sweden; ‡ Glass and Time, IMFUFA, Department of Science and Environment, Roskilde University, P.O. Box 260, DK-4000 Roskilde, Denmark; § Institut Laue-Langevin, 71 Avenue des Martyrs, CS 20156, 38042 Grenoble Cedex 9, France; ∥ Insitute of Physics, University of Silesia, 75 Pulku Piechoty 1, 41-500 Chorzow, Poland; ⊥ Department of Chemistry, Saarland University, 66123 Saarbrücken, Germany; # Diamond Light Source, OX11 0DE Didcot, United Kingdom

## Abstract

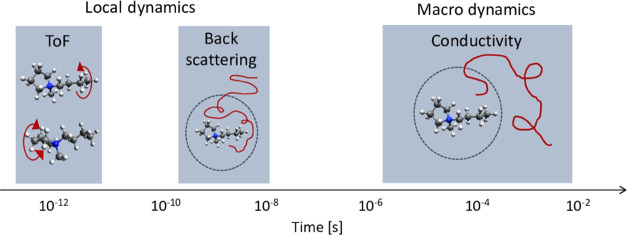

A detailed understanding of the local dynamics in ionic liquids remains an important aspect in the design of new ionic liquids as advanced functional fluids. Here, we use small-angle X-ray scattering and quasi-elastic neutron spectroscopy to investigate the local structure and dynamics in a model ionic liquid as a function of temperature and pressure, with a particular focus on state points (*P*,*T*) where the macroscopic dynamics, i.e., conductivity, is the same. Our results suggest that the initial step of ion transport is a confined diffusion process, on the nanosecond timescale, where the motion is restricted by a cage of nearest neighbors. This process is invariant considering timescale, geometry, and the participation ratio, at state points of constant conductivity, i.e., state points of isoconductivity. The connection to the nearest-neighbor structure is underlined by the invariance of the peak in the structure factor corresponding to nearest-neighbor correlations. At shorter timescales, picoseconds, two localized relaxation processes of the cation can be observed, which are not directly linked to ion transport. However, these processes also show invariance at isoconductivity. This points to that the overall energy landscape in ionic liquids responds in the same way to density changes and is mainly governed by the nearest-neighbor interactions.

## Introduction

In the last decades, there has been a rapidly growing interest in ionic liquids (ILs) for a range of different applications.^1^ With characteristic properties such as high thermal stability, a large electrochemical stability window, and low vapor pressure they have been highlighted as electrolyte components for next-generation energy-storage systems with potential to improve both safety and performance.^2,3^ For these, as well as several other applications, ion transport is in focus and it is central to understand the connection between the microscopic structure and dynamics and the ionic conductivity to design new ionic liquid electrolytes.

Ionic liquids (ILs) are salts with a melting point below 100 °C. Being constituted only of ions, Coulombic interactions are obviously of importance but there is a competition with attractive van der Waals interactions from apolar parts of the ions, e.g., alkyl side chains on the cation. As a result of the competing interactions, a particular nanoscale structure, not present in simple molecular liquids, is a hallmark of ILs.^4^ In addition to nearest-neighbor correlations, an ordering on nanometer-length scales is found as a result of charge ordering and segregation of apolar domains.^5−8^ These heterogeneities are manifested in scattering experiments, e.g., small-angle X-ray scattering (SAXS), as peaks at low momentum transfers (*Q*), typically 0.1–0.4 Å^–1^ for apolar domains and 0.6–1 Å^–1^ for charge ordering, in addition to the molecular (nearest neighbor) correlation peak found in all liquids of around 1.3–1.6 Å^–1^.

Macroscopic transport properties in ionic liquids are typically investigated by dielectric spectroscopy,^9−11^ conductivity experiments,^12−14^ or rheology,^15^ and based on these length scales, a close correlation between viscous flow and ion transport has been inferred.^16,17^ On microscopic-length scales, dynamics has been studied by, e.g., nuclear magnetic resonance,^18,19^ quasi-elastic neutron scattering (QENS),^20−28^ and molecular dynamics simulations.^29^ QENS, in particular, is a suitable probe to investigate microscopic dynamics of ionic liquids as it provides direct access to the length scales of nearest-neighbor, polar, and apolar structural correlations. Depending on the energy resolution of the QENS instrument, motions on timescales from picoseconds to nanoseconds can be followed in the momentum transfer range of 0.1–2 Å^–1^. Previous QENS experiments propose the presence of a complex landscape of dynamics with fast local processes (1–10 ps), such as methyl group rotations, alkyl side-chain relaxations, cation libration, and confined diffusion processes (10–100 ps), preceding the more long-range diffusion dynamics (1–100 ns).^20−28^ Furthermore, with neutrons as a probe, it is possible to use partial deuteration of the ions or polarized neutrons to highlight, or suppress, the contribution of dynamical processes connected to different parts of the ions.^28^

Here, we target the link between the macroscopic and microscopic dynamics of ionic liquids and the structure by investigating the temperature and pressure dependence of the structure and dynamics of a model ionic liquid. The temperature and pressure dependence of the structure in ionic liquids have previously been investigated by SAXS^6,30−32^ and molecular dynamics simulations.^7,29,33^ While the length scale of the apolar domains, defined by the position of a low *Q* peak in the static structure factor, follows the trend of the overall density change, the intensity of the peak decreases with increasing temperature and increasing pressure.^23,31−33^ With increased temperature, the driving force for segregation into apolar domains is decreased, whereas conformational changes of the alkyl side chains have been suggested to be responsible for the collapse of the domains at a high pressure, >2 GPa.^32^ The position of the charge ordering peak has also been shown to follow the density as a function of temperature but with a weaker dependence.^6,7,30^ The pressure dependence of the charge ordering correlation is not as well investigated but a shift of the peak to lower *Q*, and potentially, also the decrease in the intensity, with increasing pressure has been suggested.^32^

On macroscopic-length scales, the pressure and temperature dependence of dynamics has been investigated by, e.g., viscosity or conductivity experiments.^12,34^ As expected, the increased pressure and decreased temperature lead to a slow-down of ion transport. It has been shown that the ionic conductivity in ILs follows a similar density scaling found for molecular liquids,^35^ and we have recently shown that the overall microscopic dynamical response also obeys the same density scaling as found for transport properties.^36^ However, to the best of our knowledge, details of the microscopic dynamics, such as geometry, timescale, and the participation ratio of ions in local processes have so far not been explored in *P*–*T* space and directly connected to macroscopic ion transport.

**Figure 1:**
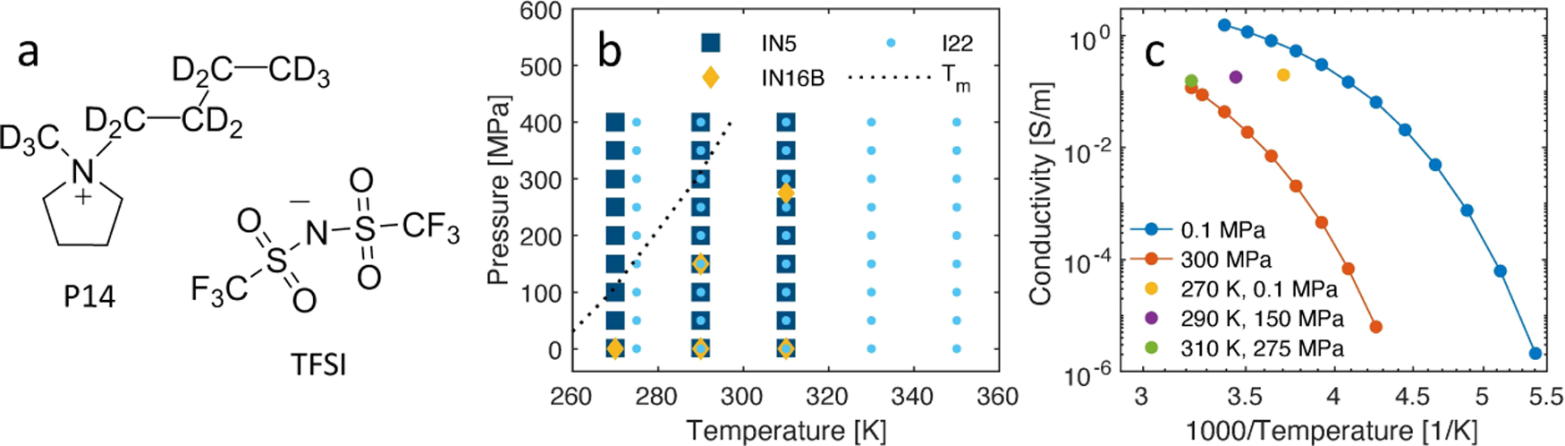
(a) Structure of partly deuterated ionic liquid P14TFSI, (b) *PT* space covered in QENS (instruments IN5 and IN16B, respectively) and SAXS (I22 instrument) experiments. The dashed line indicates the evolution of the melting temperature, *T*_m_, data from ref (38). (c) Conductivity as a function of temperature at two constant pressures and at three state points of isoconductivity.

In this work, we use SAXS, QENS, and conductivity measurements to investigate structure and micro- and macroscopic dynamics in the archetypal ionic liquid 1-butyl-1-methylpyrrolidinium bis(trifluoromethanesulfonyl)imide (P14TFSI), Figure 1a, with the aim to distinguish which local processes are linked to ion transport and their relation to the structural correlations in the ionic liquid. We focus on the liquid phase in the *PT* diagram, Figure 1b, but we also enter the supercooled regime of the ionic liquid at low temperatures and high pressures. A particular feature of the experiment is that the neutron and conductivity experiments are performed simultaneously in a custom-built cell,^37^ allowing us to directly explore the same state points with the two techniques. Of particular interest is to explore the local structure and dynamics at state points where the conductivity is constant, here called points of isoconductivity, as shown in Figure 1c. Two different QENS instruments were used to access a time window that covers both the comparatively fast and slow local dynamics in the ionic liquid. A partly deuterated ionic liquid, deuterated side chain and a methyl group on the cation (Figure 1a), was used in the QENS experiments to decrease the contribution from local dynamics (e.g., methyl group rotation or butyl side-chain dynamics) in the region of potential diffusive dynamics and to focus on the incoherent scattering from the core of the cation.

## Methods

### Materials

A partly deuterated sample, deuterated side chain and methyl group on the cation, of 1-butyl-1-methylpyrrolidinium bis(trifluoromethanesulfonyl)imide (P14TFSI), was used for the neutron-scattering experiments (Figure 1a). For the synthesis, pyrrolidine (≥99.5%, purified by redistillation) from Sigma-Aldrich (St. Louis), acetonitrile (≥99.9%, HPLC grade), acetone (99.9%), and dichloromethane (>99%) from Fisher Scientific (Nidderau, Germany), deuterated 1-bromobutane (D9, 98%) from Eurisotop (Saarbrücken, Germany), deuterated methyl iodide (D3, 99.5%) from Carl Roth (Karlsruhe, Germany), and lithium bis(trifluoromethanesulfonyl)imide (>99%) from IoLiTec (Heilbronn, Germany) were used as received. Solvents used in the synthesis were dried over molecular sieves. Identity and purity of the synthesized compounds were confirmed by multinuclear NMR spectroscopy on an AVANCE II 400 NMR spectrometer (Bruker, Billerica). The absence of halides in the final product was checked by testing with a AgNO_3_ solution. Details on the synthesis procedure are found in the Supporting Information. For the SAXS experiment, a fully protonated sample was used (Solvionic 99.9%). The samples were kept, handled, and loaded in the sample cells in an inert atmosphere.

### Quasi-Elastic Neutron Scattering

QENS experiments were performed using the backscattering spectrometer IN16B^39,40^ and the time-of-flight spectrometer IN5^41,42^ at Institute Laue-Langevin (ILL), in Grenoble, France. In the IN16B experiment, the energy resolution, accessible energy window, and useful *Q*-range were 0.75, −30 < ℏω < 30 μeV, and 0.4 < *Q* < 1.4 Å^–1^, respectively. With a wavelength of 5 Å on IN5, the energy resolution, energy window, and *Q*-range were 0.1 meV, −10 < ℏω < 2 meV, and 0.6 < *Q* < 1.95 Å^–1^, respectively. The sample was loaded into a high-pressure cell, sample thickness 0.3 mm, that allows for the pressure to be controlled within the range of 0.1–400 MPa.^37^ The pressure cell was inserted into a cryostat for independent temperature control in the range of 10–310 K. The pressure cell contains a cylindrical capacitor, which enables conductivity measurements to be simultaneously performed with the QENS experiment.^37^ The neutron data were reduced using LAMP^43^ and analyzed using DAVE.^44^ The IN16B data were grouped in *Q* to increase the statistics. Low-temperature measurements of the sample at 55 and 30 K were used as resolution functions for the analysis of IN16B and IN5 data, respectively.

### Small-Angle X-ray Scattering

SAXS experiments were performed at the small-angle X-ray scattering beamline I22 at Diamond Light Source, United Kingdom. Data were collected at 18 keV using two detectors, Pilatus P3-2M (SAXS) and Pilatus P3-2M-L (WAXS), to cover the *Q*-range 0.3 < *Q* < 1.6 Å^–1^. A P-Jump cell^45^ was used to control the temperature and pressure in the range of 275–350 K and 0.1–400 MPa, respectively. The sample was loaded in a plastic capillary and a water-filled capillary was used for background measurements. Data were reduced using DAWN software.^46^

## Results and Discussion

**Figure 2:**
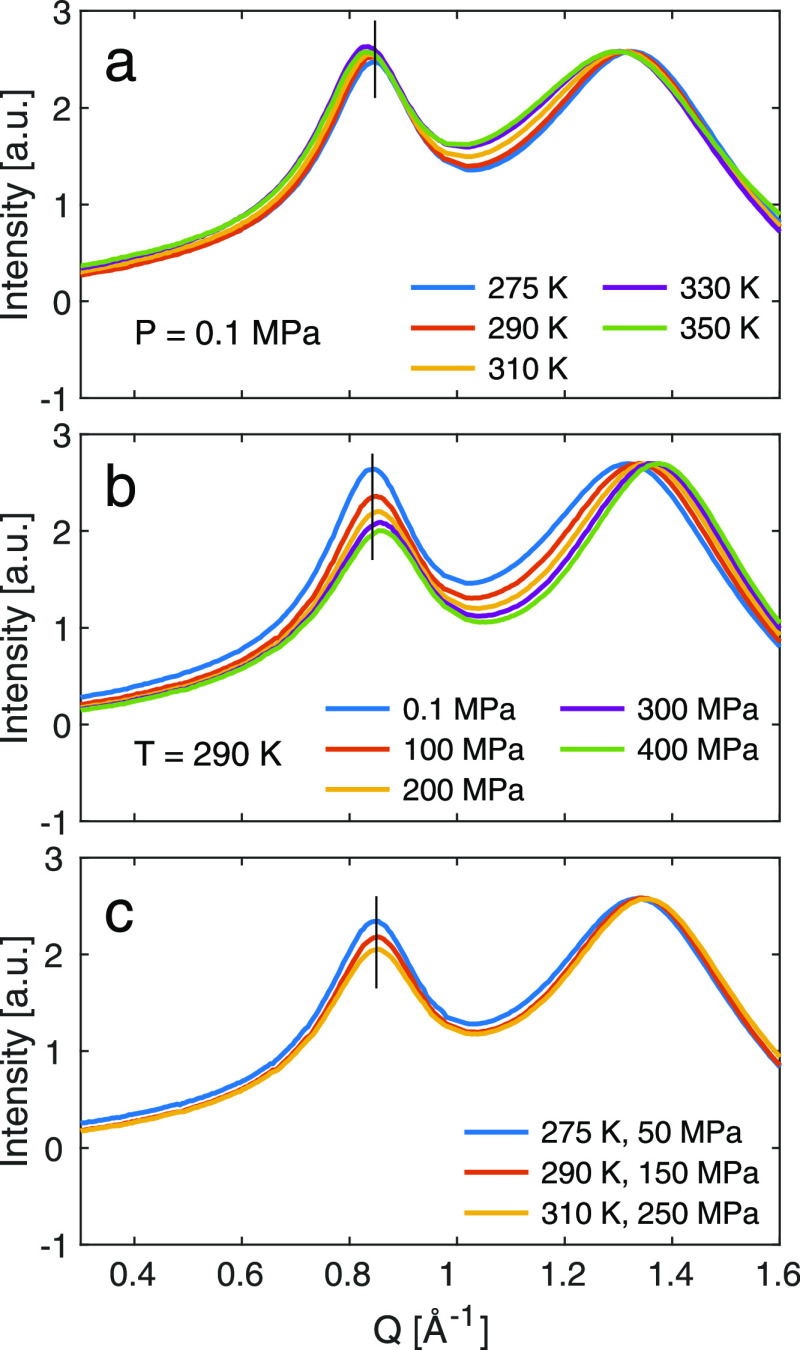
SAXS patterns for different (a) temperatures (at ambient pressure), (b) pressures (at 290 K), and (c) at state points of isoconductivity. Black lines indicate the peak position at (a) 290 K, (b) 0.1 MPa, and (c) 275 K and 50 MPa. Intensity is normalized to the height of the molecular peak.

Figure 2 shows the SAXS results for different temperatures and pressures, and in agreement with previous studies, two peaks can be observed in the SAXS pattern.^47^ At *Q* = 1.35 Å^–1^, the molecular peak reflects the nearest-neighboring distances between the ions,^48,49^ and at *Q* = 0.85 Å^–1^, the charge ordering peak arises from correlation of ions of similar charge, i.e., it accounts for the charge alteration of the ions in the liquid.^48,50^ No clear low *Q* peak related to ordering in apolar domains is observed since the side-chain length, butyl chain, is quite short, and just some excess scattering is observed at lower *Q*.

With decreasing temperature and increasing pressure, both peaks shift to higher *Q*, as shown in Figure 2a,b. Thus, overall the structural response to pressure and temperature is in line with the increase in the density of the liquid and the temperature dependence is in line with previous results.^6,7,30^ Pilar et al. showed the same pressure dependence of the molecular peak position,^32^ but in that study, the shift of the charge ordering peak was not conclusive. The position of the charge ordering peak could be expected to have weaker dependence on pressure than the molecular peak since the correlation is governed by mainly Coulombic interactions, but the possibility of conformation changes of the TFSI anion is a way to respond to compression and allow denser packing of ions. Compared to the temperature dependence, the pressure dependence of the intensity of the charge ordering peak is much stronger relative to the structural peak, pointing to a decreased ordering on the nanoscale with pressure, i.e., between similar charges, compared to nearest-neighbor correlations, i.e., dissimilar charges.

In Figure 2c, the SAXS patterns at state points of isoconductivity are compared. The three curves overlap to a large extent with respect to peak positions, whereas the intensity of the charge ordering peak decreases slightly at the state points with high pressure, reflecting the strong pressure dependence of the intensity of this peak. Thus, for state points with the same macroscopic dynamics, the structural correlations are invariant, pointing to a strong connection between the local structure and the dynamics as previously also concluded from our density-scaling study, where it was shown that the changes in the molecular peak quantitatively follow the density change.^36^ The small deviation in the overlap in the SAXS patterns at state points of isoconductivity in the range of the first peak (low *Q*) points to slight differences in the nanostructure. As this peak is related to charge ordering, we can envisage a slight reduction in the correlation between similar charges, potentially a local variation, or disorder, in the charge correlation.

**Figure 3:**
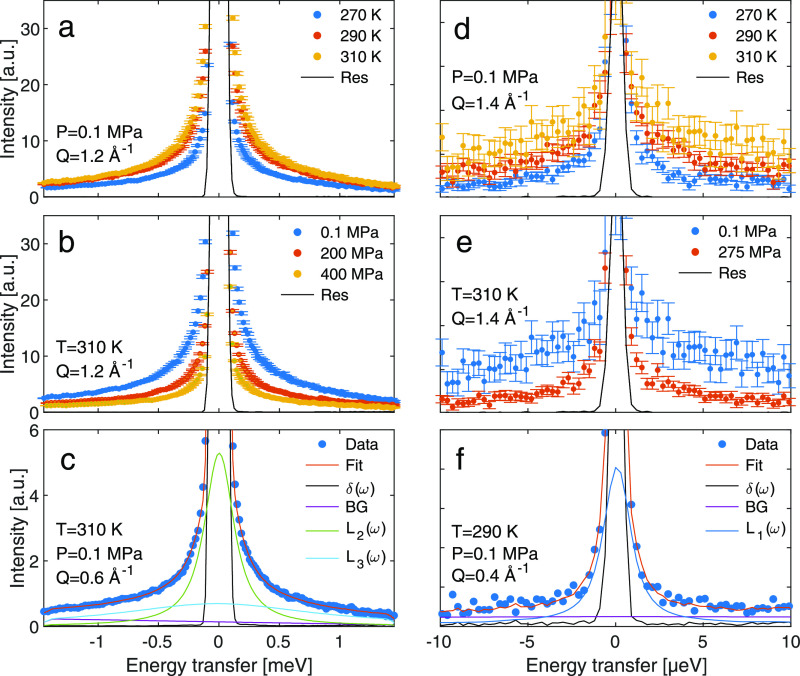
QENS spectra at different temperatures and pressures from IN5 (a) at *Q* = 1.2 Å^–1^ at 0.1 MPa and (b) at *Q* = 1.2 Å^–1^ at 310 K, and IN16B (d) at *Q* = 1.4 Å^–1^ at 0.1 MPa and (e) at *Q* = 1.4 Å^–1^ at 310 K. The intensities have been normalized to the intensity at ω = 0. (c, f) Examples of fits to the QENS data by eqs 2 and 1 for IN5 and IN16B data, respectively (see the text for details).

Turning to the dynamics, Figure 3a,b,d,e shows normalized QENS data measured at IN5 and IN16B, respectively. With decreasing temperature and increasing pressure, a slowing down of the dynamics is expected based on the behavior of the conductivity, as shown in Figure 1c. Indeed, the microscopic dynamics overall follows this trend, as observed from the narrowing of the spectra with decreased temperature and increased pressure in both experiments (Figure 3). At IN5, we follow fast dynamics, broadening of the order of 0.1–1 meV corresponding to timescales of 1–100 ps, whereas at IN16B, slower dynamics is resolved, broadening of the order of 1 μeV corresponding to timescales of 1–10 ns.

To in-detail analyze the pressure and temperature dependence of the dynamics, the data were fitted to a convolution of the resolution function of the instrument, a delta function for the elastic scattering, and Lorentzian functions for the dynamical processes, according to
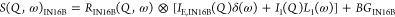
1
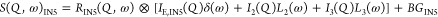
2where *R*(*Q*,ω) is the resolution function of the instrument, δ(ω) is the delta function, *L*_1_(ω), *L*_2_(ω), and *L*_3_(ω) are the Lorentzian functions, and *BG* is a linear background. *I*_E_(*Q*), *I*_1_(*Q*), *I*_2_(*Q*), and *I*_3_(*Q*) all represent the areas of the corresponding delta or Lorentzian functions. Examples of fits to the data are found in Figure 3c,f. For the slow dynamics, IN16B data, one Lorentzian function was enough to fit the data, eq 1, whereas for the fast dynamics, IN5 data, two Lorentzian functions were needed to reproduce the spectral shape, eq 2. This approach is in line with previous work investigating microscopic dynamics in ionic liquids using QENS.^20^

**Figure 4:**
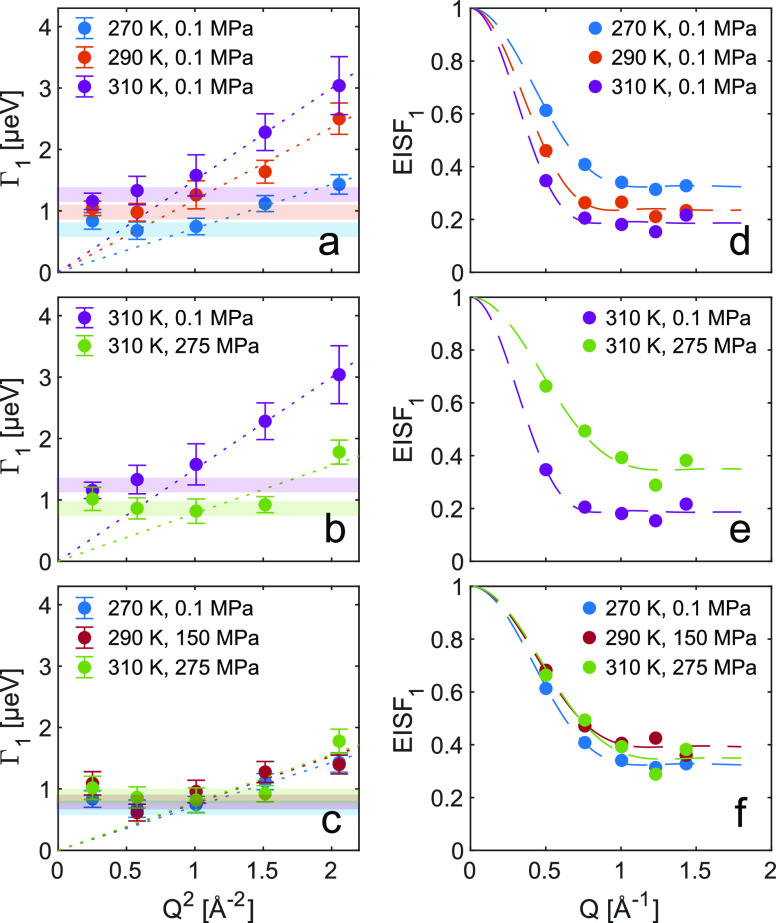
Results from fits of eq 1 to IN16B data. Momentum transfer dependence of half-width at half-maximum, Γ, and the elastic incoherent structure factor for different (a, d) temperatures, (b, e) pressures, and (c, f) at state points of isoconductivity. Dotted lines in (a–c) correspond to linear fits of Γ = ℏ*DQ*^2^ to the high *Q* region and the colored horizontal bands indicate the level of constant Γ at low *Q*. Dashed lines in (d–f) are fits to the confined diffusion model, eq 4.

The results from the fits of the slow relaxation process (IN16B) are shown in Figure 4. The half-width at half-maximum of the Lorentzian component, Γ_1_, is of the order of few μeV, which corresponds to a process on a nanoseconds timescale. Figure 4a,b shows the momentum transfer (*Q*) dependence of Γ_1_ for different temperatures at constant pressure and different pressures at constant temperature. For all pressures and temperatures, the same overall behavior is observed with a crossover from a constant value at low *Q* and to a Γ ∝ *Q*^2^ dependence at high *Q*. This is a signature of a confined diffusion process,^21,51,52^ with a diffusion coefficient, *D*, determined by Γ = ℏ*DQ*^2^. The confined nature of the motion is also reflected in the *Q*-dependence of the elastic incoherent structure factor (EISF).
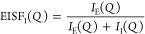
3For a confined diffusion process, the EISF can be written as^53,54^
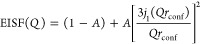
4where *j*_1_ is the spherical Bessel function of the first order, *r*_conf_ is the radius of the confinement sphere, and *A* is the participation ratio, i.e., the fraction of atoms taking part in the process.

The data in Figure 4d–f are well described by eq 4 and the parameters obtained from the fits are found in Table 1. The confinement radius, *r*_conf_, of the motion is in the range 4–5 Å, which corresponds well to the cation–anion correlation distance, and the participation ratio, *A*, falls in the range 0.7–0.8. The confinement radius can also be estimated from a relation proposed for a confined motion^52^
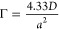
5where Γ is the constant level of the width at small *Q*, *D* is the diffusion coefficient, and *a* is the confinement radius. The values of *a*, calculated from eq 5 using the constant level of the width, as indicated in Figure 4, and the values of the diffusion coefficient, D, reported in Table 1, are in qualitative agreement, i.e., similar length scale and same pressure and temperature dependencies, with the confinement radius determined from the *Q*-dependence of the EISF, *r*_conf_, further supporting that the confinement is related to nearest-neighbor correlations.

**Table 1:**
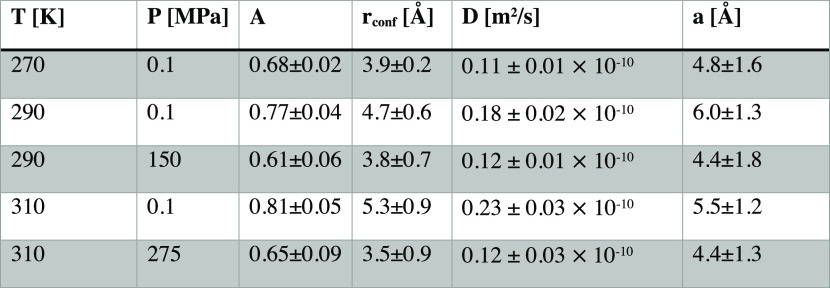
Parameters Obtained from Fitting of the *Q*-Dependence of EISF_1_ and Γ_1_, and the Estimation of the Confinement Radius, *a*, from Equation 5^a^

a State points of isoconductivity are marked in gray.

As the scattering from the cation dominates (σ_cation_ > 90% of total scattering, Table S1), the probed dynamics can be directly related to confined dynamics of the cation. The connection to cation dynamics is further underlined by the diffusion coefficients obtained from fits of the linear dependence of Γ_1_ on *Q*^2^, Table 1, which is of same order of magnitude as, but slightly higher than, previously reported data from PGSE-NMR.^55^ Thus, the confined diffusion process can be assigned to a type of caged dynamics of the cation,^56^ where the cation to diffuse first has to escape a cage of nearest neighbors, i.e., anions, and that this motion is the first step of the long-range ion transport. Faster dynamics (higher diffusion coefficient) is expected on the local scale (nanometer) probed by QENS compared to the more macroscopic scale (μm) probed by NMR, and previous work suggests up to an order of magnitude difference.^25^ We find a smaller difference between the two experiments, which can be attributed to a higher resolution in our QENS experiment, which translates to longer diffusion times and longer trajectories being probed, coming closer to the NMR experiment.

The increased pressure and decreased temperature both lead to a decrease in the confinement radius (Table 1), directly reflecting the response of the structure to density change. The diffusion coefficient follows this trend and decreases as the dynamics becomes more and more restricted and the fraction of mobile ions also decreases. The striking agreement of the data, Figure 4c,f, and parameters calculated from the fits to the data, Table 1, at state points of isoconductivity shows that the microscopic dynamics has the same nature, i.e., that the geometry of the motion, timescale, and the participation ratio are invariant, at a specific conductivity. The invariance of the confinement radius, reflecting the anion cage of the cations, points to that the long-range diffusion in this ionic liquid is, in fact, controlled by the nearest-neighbor interactions. This fact extends our previous result that the structure and the overall microscopic dynamical response is invariant at state points of isoconductivity^36^ to that also the detailed nature of the dynamics as the first step in the diffusive motion is invariant.

**Figure 5:**
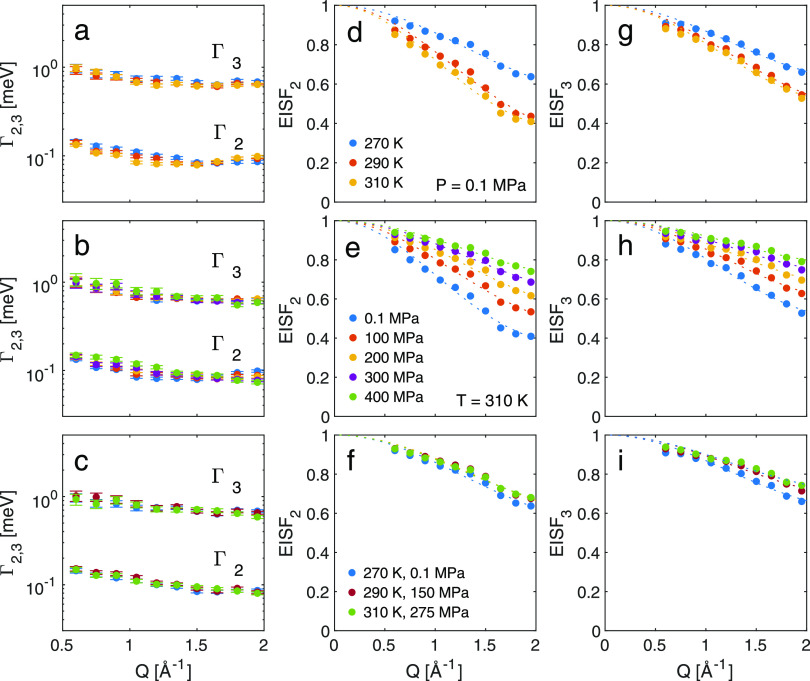
Results from fits of eq 2 to IN5 data. Momentum transfer dependence of half-width at half-maxima, Γ_2,3_ (a–c), and elastic incoherent structure factor, EISF_2,3_ (d–i) for different temperatures at ambient pressure (figures in the top row), pressures at 310 K (middle row), and at state points of isoconductivity (bottom row). Dotted lines in figures (d–f) and (g–i) are fits to eqs 4 and 8, respectively.

From the IN5 experiment, we obtain information on dynamics on shorter timescales compared to the diffusive dynamics probed with IN16B. The results from the fits of the IN5 data to eq 2 are shown in Figure 5. The half-widths at half-maximum (Γ_2_, Γ_3_) of the two processes obtained from the fits are in the order of meV and the timescales for the two motions can be estimated as 6–40 ps, respectively. The widths of the two processes show no *Q*-dependence (Figure 5a,b), suggesting that these are localized relaxations. The slight increase of Γ at lower *Q* values can be attributed to a partial coherent contribution to the scattering, as proposed in previous studies.^57^ One can also note that both processes virtually show no pressure or temperature dependence. This is in line with previous results on an imidazolium-based ionic liquid, where the widths of the corresponding processes showed a very weak temperature dependence and no *Q*-dependence.^20^ Here, we show that this behavior is also valid as a function of pressure.

To further investigate the nature of the two relaxations, we calculate the EISFs according to eqs 6 and 7.
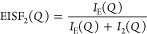
6
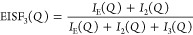
7For the slower process (*L*_2_ in eq 2), EISF_2_ is calculated in the same way, eq 6, as for the single process in the IN16B data, taking into account the pure elastic scattering, *I*_E_, with respect to the sum of the elastic scattering and the slow quasi-elastic process (*I*_2_). The faster process is here considered as a background. For the faster process (*L*_3_ in eq 2), the intensity of the slower process (*I*_2_ of the narrow component in Figure 3c) is treated as an elastic contribution, as shown in eq 7. This approximate approach can be justified as a result of the relatively large separation in time between the two processes. Figure 5d–i shows the two functions together with fits to eq 4 (EISF_2_), which, in this case, represents a restricted relaxation rather than a confined diffusion, following the approach previously applied for ionic liquids,^20^ and to eq 8 (EISF_3_) for a circular rotation motion, following the approach proposed in ref (20).
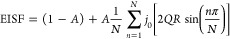
8It describes random jumps among *N* equivalent sites on a circle with radius *R*. For large *N*, this is equivalent to a continuous rotational diffusion.

The parameters obtained from the fits are found in Table 2. For the slower relaxation process, a radius of around 1.3–1.6 Å is found, which can be associated to a librational motion of the ring of the cation since this length scale would not allow a full rotation of the ring.^20,51^ The confinement radius of this librational motion, as well as the ratio of atoms participating in the motion, *A*, decreases with decreasing temperature and increasing pressure. From the fits of eq 8 to EISF_3_ (the faster process), a rotation radius ∼1 Å is obtained. This length scale can be assigned to the relaxation, e.g., conformational change, of the butyl side chain of the cation following similar results for an imidazolium-based ionic liquid.^20^ Even though the butyl side chain is deuterated, its dynamics can be picked up as there is still an incoherent scattering contribution from deuterium, which is of the same order as the coherent scattering. The rotation radius shows no pressure or temperature dependence, but the participation ratio decreases with decreasing temperature and increasing pressure. A decrease in the participation ratio with decreasing temperature for a local process, such as the librational motion, is in agreement with previous results from QENS experiments on ionic liquids.^58^ It was there inferred that the local dynamics was gradually frozen out as temperature decreases, i.e., that there is a heterogeneity in the dynamics and at one instant moment not all ions are mobile. Here, we show that increasing pressure has the same effect in gradually freezing out the local dynamics.

**Table 2:**
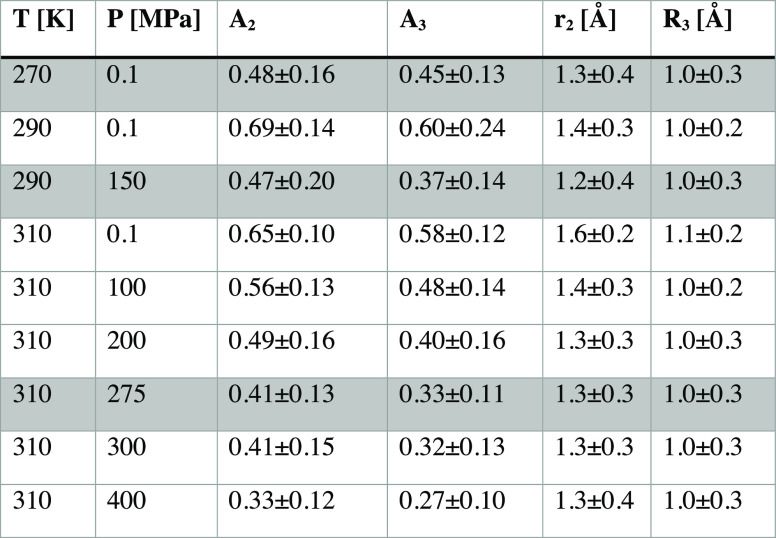
Parameters from Fitting EISF_2_ to Equation 4 and EISF_3_ to Equation 8^a^

a State points of isoconductivity are marked in gray.

A comparison of the parameters for the two fast localized relaxations obtained at state points of isoconductivity reveals that the geometry of both motions is invariant. However, the processes are not fully invariant at isoconductivity as the participation ratios show a slight decrease with pressure, pointing to the fact that pressure has a stronger influence on the number of ions taking part in the relaxation. This deviation of invariance for the participation ratio can potentially be correlated to the small difference in charge ordering observed in the SAXS patterns, Figure 2c, with pressure pointing to a local variation in the nanostructure at state points of isoconductivity that also influences the local dynamics. Even though these two localized processes show some invariance at constant conductivity, we do not believe that they are part of the conduction process due to the lack of temperature and pressure dependence of the width (timescale) of the two processes.

## Conclusions

**Figure 6:**
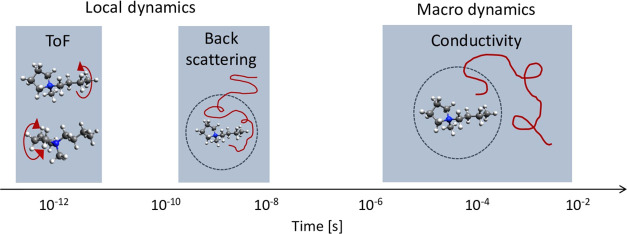
Schematic of the microscopic and macroscopic dynamics in P14TFSI.

We have investigated the structure and microscopic dynamics of an ionic liquid as a function of temperature and pressure. In our analysis, three cation relaxations are revealed, as shown in Figure 6, and assigned to confined translational diffusion of the cation (nanoseconds timescale), restricted local librational motion of the ring of the cation (timescales around 6 ps), and cation side-chain relaxation, e.g., conformational change/segmental rotation (timescales around 40 ps), respectively. The diffusion constant calculated for the slow, nanosecond, process corresponds well to the macroscopic diffusion of the cation measured by NMR, a result that provides a direct link between the microscopic and macroscopic dynamics. This is also underlined by the fact that the process (both the geometry and the participation ratio) is fully invariant at state points of isoconductivity. The nature of the diffusive process can be seen as a confinement of the cation in a cage by nearest neighbors (anions), suggesting that the rate-limiting step for ion transport is cage dynamics. This is also supported by the invariance of the nearest-neighbor correlations in the SAXS data at isoconductivity, whereas the charge ordering correlation on longer length scales shows some deviations. For the two faster-localized motions, their geometry is also invariant at isoconductivity, which is reasonable, considering that the local environment is determined by nearest neighbors. However, the lack of any temperature or pressure dependence of the timescale of the motion excludes a direct connection between this type of dynamics and the macroscopic ion transport.

To the best of our knowledge, this is the first experimental investigation on how the pressure affects the detailed nature of the microscopic processes in ionic liquids, as well as using the concept of isoconductivity to better understand the relation between the microscopic and macroscopic properties. The result that all processes are invariant at state points of isoconductivity is fully in line with isomorph theory for liquids, which predicts this invariance without claiming that there is a causal connection between different relaxations.^59^ The invariance can rather be interpreted as that it is the whole energy landscape that scales in the same way with density. We believe that the approach taken in this study, investigating both the pressure and temperature dependence of the structure and the microscopic dynamics, is a viable route to further build the understanding between a microscopic structure and dynamics and ion transport in ionic liquids, as well as in other highly concentrated electrolyte systems.

## Supporting Information Available

Neutron-scattering cross sections and the description of the synthesis of partly deuterated 1-butyl-1-methylpyrrolidinium bis(trifluoromethanesulfonyl)imide (PDF)
