# Supplementary material for: Pressure and Temperature Dependence of Local Structure and Dynamics in an Ionic Liquid
Source: J Phys Chem B. 2021 Mar 3;125(10):2719–28. doi: 10.1021/acs.jpcb.1c00147 (PMC8034775; doi:10.1021/acs.jpcb.1c00147)
Supplement: Supplementary file 1 — jp1c00147_si_001.pdf [file jp1c00147_si_001.pdf]

SUPPORTING INFORMATION

Pressure and Temperature Dependence of Local Structure and Dynamics in an Ionic Liquid

Filippa Lundin[1], *Henriette Wase Hansen*[1,2,3], *Karolina Adrjanowicz*[4], *Bernhard Frick*[3], *Daniel Rauber*[5], *Rolf Hempelmann*[5], *Olga Shebanova*[6], *Kristine Niss*[2], *and Aleksandar Matic*[*1]

[1]Department of Physics, Chalmers University of Technology, SE-41296 Göteborg, Sweden

[2]Glass and Time, IMFUFA, Department of Science and Environment, Roskilde University, Postbox 260, DK-4000 Roskilde, Denmark

[3]Institut Laue-Langevin, 71 avenue des Martyrs, CS 20156, 38042 Grenoble Cedex 9, France

[4]Insitute of Physics, University of Silesia, 75 Pulku Piechoty 1, 41-500 Chorzow, Poland

[5]Department of Chemistry, Saarland University, 66123 Saarbrücken, Germany

[6]Diamond Light Source, Didcot OX11 0DE, United Kingdom

NEUTRON SCATTERING CROSS SECTIONS

Table S1. Neutron scattering cross sections for the ionic liquid P14TFSI.

Component	Chemical formula	σ_{coh} [barn]	σ_{inc} [barn]
Anion	$C_2F_6NO_4S_2$	65.18	0.54
Cation – partially deuterated	$C_9H_8D_{12}N$	142.24	667.06
P14TFSI – partially deuterated	$C_{11}H_8D_{12}F_6N_2O_4S_2$	207.42	667.7

SYNTHESIS PROCEDURE OF DEUTERATED P14TFSI

SYNTHESIS OF 1-(D9-BUTYL)-PYRROLIDINE

For the synthesis of the title compound 28.3 mL of pyrrolidine (24.4 g; 342 mmol; 5.0 eq.) were dissolved in 200 mL of dry acetonitrile under argon atmosphere. The mixture was cooled with an ice bath and 7.84 mL of fully deuterated 1-bromobutane (10 g; 68.5 mmol; 1.0 eq.) were added by dropwise addition over two hours. The mixture was allowed to warm to ambient temperature and stirred for five days under these conditions. The solvent was removed by rotary evaporation and the residue was subjected to fractional distillation to remove the excess of pyrrolidine. The product (1-(d9-butyl)-pyrrolidine) was obtained in 70% yield (6.54 g; 48.1 mmol) as colorless liquid (boiling point of 156°C at 1.013 mbar).

SYNTHESIS OF 1-(D9-BUTYL)-1-(D3-METHYL)-PYRROLIDINIUM IODIDE

6.20 g of (1-(d9-butyl)-pyrrolidine (45.6 mmol; 1.0 eq.) were dissolved in 200 mL of dry acetonitrile under an inert atmosphere of argon and cooled with an external ice bath. To the homogenous solution 3.41 mL of fully deuterated methyl iodide (7.77 g; 54.7 mmol; 1.2 eq.) was added dropwise over three hours. The solution was allowed to heat to ambient temperature and stirred in the water bath for three days. The excess of reagents and the solvent were removed in vacuum and the obtained solid product further dried in high vacuum. The title compound was obtained in 98% yield (12.4 g; 44.7 mmol) as a colorless solid.

SYNTHESIS OF 1-(D9-BUTYL)-1-(D3-METHYL)-PYRROLIDINIUM BIS(TRIFLUOROMETHANESULFONYL)IMIDE

11.5 g of 1-(d9-butyl)-1-(d3-methyl)-pyrrolidinium iodide (41.5 mmol; 1.0 eq.) were dissolved in 200 mL of dry acetone under argon atmosphere. To the homogenous solution 14.3 g of lithium bis(trifluoromethanesulfonyl)imide (49.8 mmol; 1.2 eq.) were added. The solution was stirred for 24 hours and the solvent removed by rotary evaporation. The residue was dissolved in 250 mL dichlormethane and the suspension filtered. The organic phase was subsequently washed four times with about 50 mL of deionized water. Absence of residual iodide in the wash water was confirmed by testing with silver nitrate solution. The solvent was removed by

rotary evaporation and the residue dried in high vacuum with stirring for three days. The product was obtained in 97% yield (17.5 g; 40.3 mmol) as a colorless, low-viscous liquid.
